# Comparative Analysis of Pentacyclic Triterpenic Acid Compositions in Oleogum Resins of Different *Boswellia* Species and Their In Vitro Cytotoxicity against Treatment-Resistant Human Breast Cancer Cells

**DOI:** 10.3390/molecules24112153

**Published:** 2019-06-07

**Authors:** Michael Schmiech, Sophia J. Lang, Katharina Werner, Luay J. Rashan, Tatiana Syrovets, Thomas Simmet

**Affiliations:** 1 Institute of Pharmacology of Natural Products and Clinical Pharmacology, Ulm University, 89081 Ulm, Germany; michael.schmiech@uni-ulm.de (M.S.); sophia.lang@uni-ulm.de (S.J.L.); katharina.werner@uni-ulm.de (K.W.); tatiana.syrovets@uni-ulm.de (T.S.); 2 Medicinal Plants Division, Research Center, Dhofar University, Salalah 211, Oman; luayrashan@yahoo.com

**Keywords:** frankincense, *Boswellia*, *Boswellia occulta*, pentacyclic triterpenic acid, boswellic acid, lupeolic acid, breast cancer, MDA-MB-231

## Abstract

Pentacyclic triterpenic acids from oleogum resins of *Boswellia* species are of considerable therapeutic interest. Yet, their pharmaceutical development is hampered by uncertainties regarding botanical identification and the complexity of triterpenic acid mixtures. Here, a highly sensitive, selective, and accurate method for the simultaneous quantification of eight boswellic and lupeolic acids by high-performance liquid chromatography with tandem mass spectrometry detection (HPLC-MS/MS) was developed. The method was applied to the comparative analysis of 41 oleogum resins of the species *B. sacra*, *B. dalzielli*, *B. papyrifera*, *B. serrata*, *B. carterii*, *B. neglecta*, *B. rivae*, *B. frereana*, and *B. occulta*. Multivariate statistical analysis of the data revealed differences in the triterpenic acid composition that could be assigned to distinct *Boswellia* species and to their geographic growth location. Extracts of the oleogum resins exhibited cytotoxicity against the human, treatment-resistant, metastatic breast cancer cell line MDA-MB-231. Extracts from *B. sacra* were the most potent ones with an average IC_50_ of 8.3 ± 0.6 µg/mL. The oleogum resin of the *B. sacra* was further fractionated to enrich different groups of substances. The cytotoxic efficacy against the cancer cells correlates positively with the contents of pentacyclic triterpenic acids in *Boswellia* extracts.

## 1. Introduction

Frankincense, an oleogum resin from trees of the genus *Boswellia* Roxb. ex Colebr. of the *Burseraceae* family, is widely used in traditional Arab, African, Ayurvedic, and Chinese medicines to treat various ailments including fever, pain, and swelling [1,2,3,4]. Several clinical trials provided some evidence for potential therapeutic efficacy of frankincense in asthma, rheumatoid arthritis, Crohn’s disease, osteoarthritis, and collagenous colitis [5]. Moreover, several pilot studies indicate possible benefits of *Boswellia* extract treatment in cancer patients [6]. Hence, identification of biologically active compounds from *Boswellia* oleogum resins would facilitate their rational exploration for therapeutic applications.

Frankincense trees grow mainly in dry areas of East Africa, the Arabian Peninsula, and in India. The trees have a shrubby appearance and a height of usually 2 –6 m, with just a few of them reaching up to 10 m [1]. The main representative species are Boswellia sacra Flueck (Oman), Boswellia carterii Birdw. (Somalia), and Boswellia serrata Roxb. ex Colebr. (India). The chemical composition of oleogum resins from these species has been extensively studied [7,8,9,10,11,12,13,14]. Likewise, the oleogum resin of Boswellia papyrifera Hochst., abundant in Ethiopia and Sudan, has been investigated regarding its chemical constituents [13,15]. Yet, there are also less known species like *Boswellia dalzielli* Hutch. that are more prevalent in West Africa, *Boswellia neglecta* S. Moore, which is abundant, for example in Kenya, or the recently discovered *Boswellia occulta* Thulin, DeCarlo & S. P. Johnson, distributed in a small area of northwestern Somalia [1,2,16]. There are about twenty-five different *Boswellia* species, but this number might include some double-counted species [2]. The geographical distribution of various *Boswellia* species often overlap, necessitating correct plant identification. However, a proper botanical classification of plant material is often hardly possible, because of their growth taking place in less developed and geopolitically unstable countries, such as Yemen, Somalia, Ethiopia, or Sudan. Hence, less is known about chemical composition and potential therapeutic value of oleogum resin ingredients from *Boswellia* species grown in these areas.

The oleogum resin is evolving after coagulation and drying of a sticky-milky liquid emerging from incisions in the bark of trunk and branches. *Boswellia* oleogum resins contain 15–20% boswellic and lupeolic acids, pentacyclic triterpenic acids (PTA) [10]. These PTA are believed to be the active principle of frankincense and have been shown to modulate pathogenetic essential pathways of inflammatory diseases and cancer [3,6,10,17,18,19,20,21,22]. Thus, boswellic acids inhibit gene expression of proinflammatory cytokines through interaction with IκB kinases [20,21]. Likewise, they inhibit 5-lipoxygenase and leukotrienes biosynthesis [17,23]. Furthermore, it has been reported that β-boswellic acid and acetyl-11-keto-β-boswellic acid are capable of inhibition of the human protease cathepsin G [24]. Another PTA, acetyl-lupeolic acid, inhibits AKT kinase crucial for tumor growth [18]. Moreover, PTA induce apoptosis in various cancer cell lines, such as brain, leukemia, breast, prostate [18,21,25,26,27] and inhibit topoisomerases [19] pointing to their anticancer properties.

To further study the pharmacotherapeutic potential of *Boswellia* oleogum resin extracts, we have developed and validated a HPLC-MS/MS method for simultaneous, selective, and highly sensitive detection of eight different PTA, boswellic and lupeolic acids. We have characterized the PTA contents in frankincense extracts from nine different *Boswellia* species grown in different localities of the Arabian Peninsula, in Africa, and India. The contents of individual PTA were quantified, evaluated by multivariate statistical methods, and correlated to the extract’s toxicity towards highly metastatic triple negative breast cancer cells (MDA-MB-231). The aim of the study was to discover patterns in the PTA composition of *Boswellia* oleogum resins of different species and to explore correlations between individual PTA contents and cytotoxic efficacies against cancer cells.

## 2. Results

### 2.1. Extraction of Boswellia Oleogum Resins

**Figure 1:**
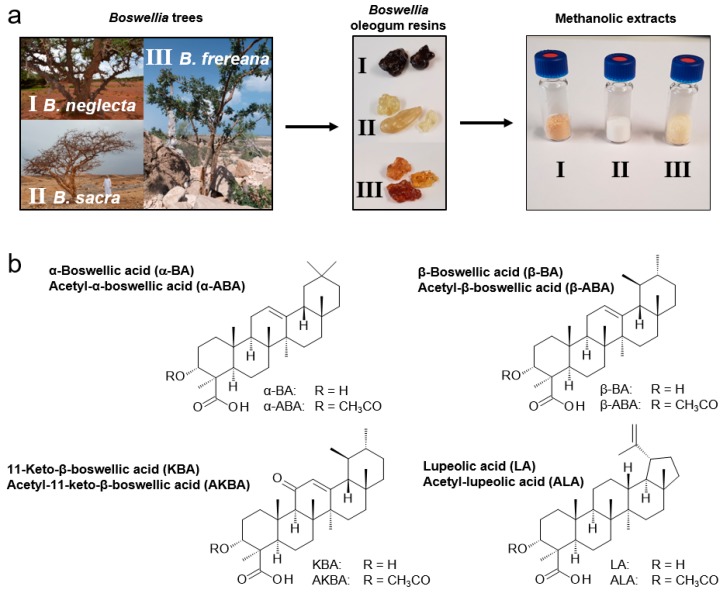
Preparation of extracts from *Boswellia* oleogum resins and chemical structures of major pentacyclic triterpenic acids (PTA). (**a**) Three *Boswellia* species, *B. neglecta* (I), *B. sacra* (II), and *B. frereana* (III) (with permission from Georg Huber, https://weihrauch-blog.de/bilder/), their oleogum resins (frankincense), and methanolic extracts thereof. (**b**) Structures of eight pentacyclic triterpenic acids (PTA) present in frankincense.

**Figure 2:**
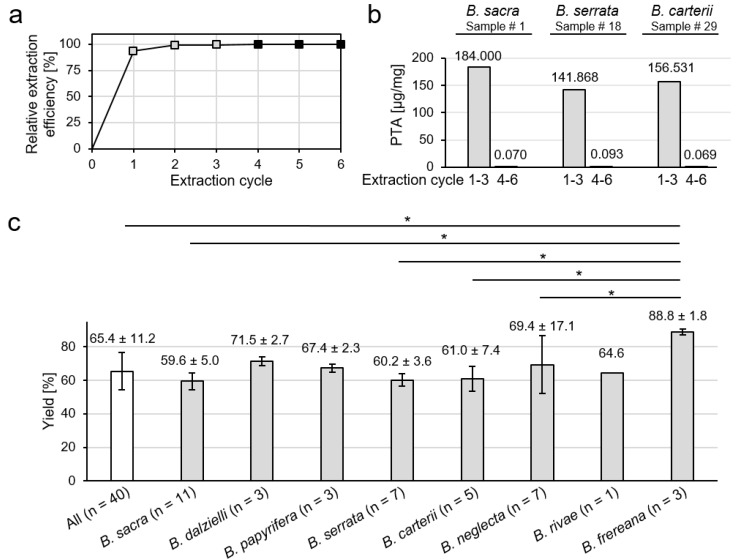
Methanolic extraction of *Boswellia* oleogum resins. (**a**) Average extraction efficacy obtained from sample # 1 (*B. sacra*), sample # 18 (*B. serrata*), and sample # 29 (*B. carterii*) with increasing extraction cycles. (**b**) Total amount of pentacyclic triterpenic acids (PTA) extracted from oleogum resins in µg/mg frankincense. Comparison of extraction efficacies of combined extraction cycles 1 –3 and 4–6. (**c**) Comparison of the extraction yields (*w*/*w*) of different *Boswellia* species. Data are mean ± SD. Groups were compared by Kruskal-Wallis one-way analysis of variance and the Mann-Whitney U test (* *p* < 0.05).

The extracts were obtained in a crystalline form (Figure 1a). The color of the extracts varied from white (*B. sacra*) and yellow (*B. frereana Birdw.*) to light brown (*B. neglecta*). To preserve all ingredients including pentacyclic triterpenic acids (Figure 1b), the extraction was carried out at room temperature. In previous studies, we have shown that methanol is a very efficient solvent for the extraction of frankincense [10]. It ensures optimal solubility of the extracts and is, therefore, used in sample analysis. To investigate, how many extraction cycles are necessary for an exhaustive extraction, three samples were extracted up to six times, each in triplicates. The experiment showed that the first three extraction cycles have an extraction efficiency of 99.6% (Figure 2a). Moreover, 99.95% of the available PTA were extracted within the first three extraction cycles (Figure 2b).

For comparative analysis, forty samples of mainly commercially available *Boswellia* oleogum resins (samples # 1–40) were extracted for analysis of their PTA contents and cancer cell toxicities. The average extraction yield was 65.4% (*w*/*w*). Extracts of *B. frereana* showed a significantly higher average yield of 88.8% (*w*/*w*) compared to the overall average value, as well as compared to *B. sacra*, *B. serrata*, *B. carterii*, and *B. neglecta* (Figure 2c). The higher extraction yields can be explained by a lower percentage of gum in *B. frereana* oleogum resins, which is slightly soluble in alcohols [1].

### 2.2. Analysis of Pentacyclic Triterpenic Acids in Boswellia Extracts

**Figure 3:**
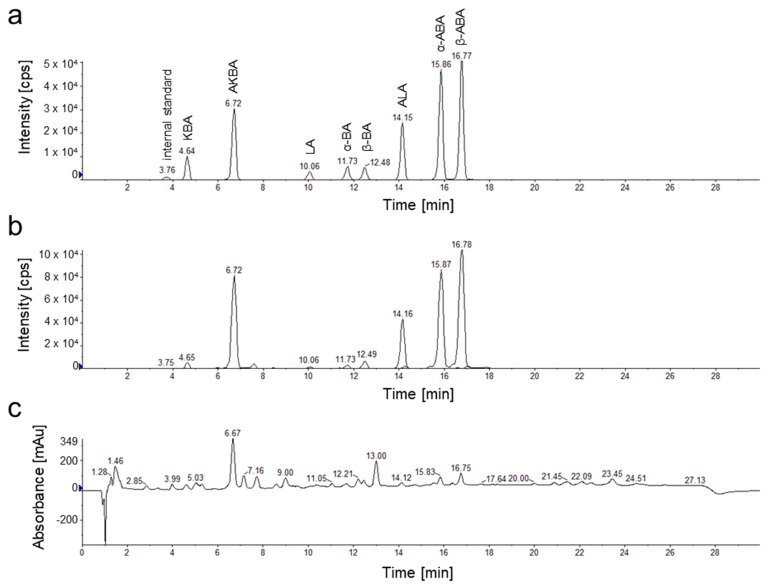
HPLC-DAD-MS/MS chromatograms of reference substances and a frankincense extract of *B. sacra*, Superior Hojari (sample # 1). (**a**) Multiple reaction monitoring chromatogram of eight pentacyclic triterpenic acids (PTA) and maslinic acid used as an internal standard. (**b**) Multiple reaction monitoring chromatogram of a *B. sacra* extract. (**c**) Total wavelength chromatogram (210 nm, 254 nm, and 280 nm) of a *B. sacra* extract.

For chemical characterization of the frankincense extracts, eight pentacyclic triterpenic acids (PTA), α-boswellic acid (α-BA), acetyl-α-boswellic acid (α-ABA), β-boswellic acid (β-BA), acetyl-β-boswellic acid (β-ABA), 11-keto- β-boswellic acid (KBA), acetyl-11-keto-β-boswellic acid (AKBA), lupeolic acid (LA), and acetyl-lupeolic acid (ALA), were quantified by HPLC-MS/MS analysis. Since PTA have very similar structures, they exhibit similar physical properties (Figure 1B). Thus, PTA are highly lipophilic. Furthermore, α-BA, β-BA, and LA, as well as ALA, α-ABA and β-ABA are constitutional isomers and, thus, have identical molecular masses. Our HPLC method, still, separates PTA selectively by a reversed-phase C18 column. To enable quantification of individual PTA even in complex biological matrices like oleogum extracts, we developed a selective and sensitive detection method (Figure 3). Detection by tandem mass spectrometry in multiple reaction monitoring mode (MRM) enabled sensitive quantification with limits of detection between 0.4 and 1.6 ng/mg (PTA/extract, *w*/*w*). Recoveries between 91.2% and 99.8% confirmed that the method allows precise quantification of the analytes in complex matrices (Table 1).

To discover differences regarding the PTA composition in frankincense, we extracted oleogum resins from different *Boswellia* species and different geographic growth locations (sample # 1–40) and analyzed the extracts using HPLC-MS/MS. The results showed some major differences between the samples. The total percentage of PTA varies from 0% (<LOD/LOQ) in *B. frereana* (samples # 38–40) and >35% in *B. sacra* (sample # 5) and *B. carterii* (sample # 26) (see Table S1). Further, the results of the HPLC-MS/MS analysis of the extracts were corrected by the extraction yield to obtain the PTA concentrations in the corresponding oleogum resins.

### 2.3. Differences in Pentacyclic Triterpenic Acid Composition of Various Boswellia Oleogum Resins

The results of the HPLC-MS/MS analysis revealed a remarkable variety in different PTA contents in *Boswellia* species obtained from different growth localities (Table 2). Oleogum resins of the species *B. sacra* from Oman are characterized by a high proportion of the acetylated PTA, α-ABA, β-ABA AKBA, and ALA. In contrast, resins of the species *B. serrata* from India are characterized by a high proportion of the deacetylated PTA, α-BA, β-BA, KBA, and LA.

The PTA composition of the species *B. papyrifera* abundant in north-eastern corner of Africa (Ethiopia, Eritrea, and Sudan) is similar to the PTA composition of *B. sacra*. Likewise, the species *B. dalzielli* abundant in West Africa (Burkina Faso, Nigeria, and Senegal) show similarity to *B. sacra*, but exhibit higher levels of the PTA with keto groups, KBA and AKBA. Thus, the oleogum resins of the species *B. dalzielli* have the highest levels of the pharmacologically highly interesting boswellic acid AKBA [19,20,21,22] with an average concentration of 64.7 µg/mg resin. By contrast, the species *B. sacra* shows an average AKBA concentration of only 31.3 µg/mg and the species *B. serrata* even shows only 11.8 µg/mg. In resins of the species *B. frereana* from Somalia, no PTA could be detected at all. These data confirm a previous study demonstrating that *B. frereana* from Somalia contains no boswellic and lupeolic acids [12].

Resins of the species *B. neglecta* from Kenia and *B. rivae* Engl. abundant in the Ogaden region of Ethiopia are characterized by a rather low concentration of PTA, similar to *B. frereana*. However, the oleogum resins of the species *B. neglecta* and *B. rivae* still could be clearly distinguished from those of *B. frereana* based on their appearance. Thus, whilst the species *B. neglecta* and *B. rivae* produce hard, dark brown oleogum resins, the resins of *B. frereana* are more tender and of a yellow color (Figure 1a). Also, the preferred habitat of these *Boswellia* species are different. In contrast to most other species, *B. frereana* prefers higher altitudes and rocky terrains [1].

**Figure 4:**
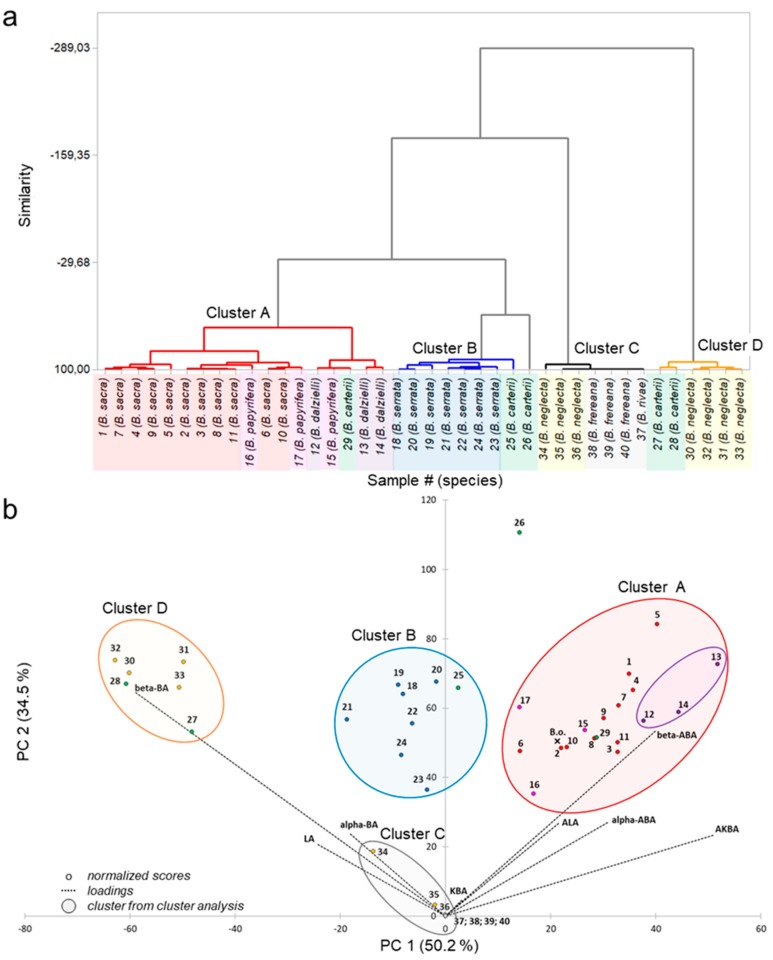
Multivariate statistical analysis of pentacyclic triterpenic acid (PTA) concentrations in *Boswellia* oleogum resins. Samples # 1–11 (*B. sacra*, red), samples # 12–14 (*B. dalzielli*, violet), samples # 15–17 (*B. papyrifera*, pink), samples # 18–24 (*B. serrata*, blue), samples # 25–29 (*B. carterii*, green), samples # 30–36 (*B. neglecta*, yellow), sample # 37 (*B. rivae*, grey), and samples # 38–40 (*B. frereana*, grey). For further sample information refer to Table 2. (**a**) Dendrogram of cluster analysis. The samples were assigned to four different clusters: cluster A (red), cluster B (blue), cluster C (grey), and cluster D (yellow). (**b**) Biplot of principal component analysis (PCA) with clusters from cluster analysis and subgroup (violet) for *B. dalzielli*. Sample B.o. (ʹXʹ B.o., *B. occulta*) was added subsequently.

To visualize the data and to explore patterns of the PTA composition, the multivariate statistical methods, principal component analysis (PCA), and cluster analysis were applied to the data set. The cluster analysis combined samples with high similarities in the PTA composition into groups, so-called clusters (Figure 4a). Furthermore, PCA combined the individual PTA concentrations to principal components by eigenvalue decomposition of the data covariance matrix. The principle components PC1 (eigenvalue percentage of 50.2%) and PC2 (eigenvalue percentage of 34.5%) are the basis for a new coordinate system of a two-dimensional subspace (biplot), in which all frankincense samples (scores) as well as the impacts of the PTA concentrations (loadings) are illustrated (Figure 4b). The cluster analysis assigned the frankincense samples to four different clusters, A-D. In combination with these clusters, the biplot of the PCA gave us information about similarity as well as the composition of the frankincense samples and disclosed hidden patterns of the data set.

Cluster A includes the species *B. sacra*, *B. dalzielli*, and *B. papyrifera* and is characterized by a high proportion of acetylated PTA. Interestingly, sample # 29 (*B. carterii*) is also included in this cluster due to similarity. For further distinction, PC3 that has an eigenvalue percentage of 11.5% and is mainly influenced by the AKBA concentrations was examined. Here, sample # 29 (*B. carterii*) and samples # 12–14 (*B. dalzielli*), as well as sample # 15 (*B. papyrifera* from Ethiopia) clearly deviated from samples of *B. sacra*. Due to the very high concentrations of AKBA in oleogum resins of *B. dalzielli*, these samples were assigned to a particular subgroup. Cluster B contains all B. serrata samples as well as sample # 25 (*B. carterii*) and it is identified by a high proportion of deacetylated PTA. With respect to PC3, sample # 25 clearly differs from samples of *B. serrata* by its very low concentrations of AKBA and KBA. Cluster C includes all samples with very low PTA concentrations, such as *B. frereana*, *B. rivae*, and *B. neglecta* from Kenia and the Bakool region in the south of Somalia. Cluster D is characterized by a very high proportion of deacetylated PTA in coincidence with a very low proportion of PTA with keto groups, such as LA, α-BA, and β-BA. This cluster contains samples of the species *B. neglecta* from Somalia and *B. carterii* from Somalia. Sample # 26 (*B. carterii*) could not be assigned to any cluster and differs from all other samples, especially due to its very high amounts of ALA and β-ABA.

Among botanists, it is intensely discussed whether *B. sacra* and *B. carterii* might be the same species, because of the similar appearance of the *Boswellia* trees, the oleogum resins, and the close geographical proximity of the provenance regions [2,13,28]. Interestingly, it has previously been reported that it is not possible to distinguish *B. carterii* from *B. sacra* analytically by HPLC [11,12]. Yet in our study, only one of five *B. carterii* samples (sample # 29) showed similarity to *B. sacra* (see cluster A), the other four *B. carterii* samples differed considerably from the *B. sacra* samples. Generally, the samples of the species B. carterii showed a major diversity among each other and no explicit pattern in the PTA composition could be discovered to characterize them. Hence, we could clearly distinguish *B. sacra* from *B. carterii* based on their PTA composition. Thus, the method developed here allows more sensitive analysis of PTA and a better differentiation of *Boswellia* species.

### 2.4. Classification of Frankincense Sampleas by the Boswellia Index (Bos_i_)

Information about the specimen’s growth locality and identification of the correct species of commercial frankincense are often insufficient. On the basis of the analytical and statistical data, we have created a formula that enables classification of frankincense samples. Hence, the concentrations of just three PTA are required to define a characteristic index, which we called the *Boswellia* index (Bos_i_):

(1) $${\mathrm{Bos}}_{i}=\left(\left[\mathrm{AKBA}\right]-1\right)\times \frac{\left[\mathrm{ABA}\right]+1}{\left[\mathrm{BA}\right]+1}\times \left(\left[\mathrm{AKBA}\right]+\left[\mathrm{BA}\right]+\left[\mathrm{ABA}\right]+1\right)\times \left(\left[\mathrm{ABA}\right]-\left[\mathrm{BA}\right]\right)$$

where [AKBA] is the concentration of AKBA in µg/mg resin, [ABA] is the concentration of β-ABA in µg/mg resin, and [BA] is the concentration of β-BA in µg/mg resin.

The Bos_i_ could be used as a tool to assist identification of *Boswellia* species and to locate the geographical growth locality of unknown frankincense samples. Calibration standards for determination of the required concentrations are commercially available. Thus, samples of the species *B. serrata* (India) from the cluster B exhibit Bos_i_ between −21,000 and −2900. Samples of the species *B. sacra* (Oman), *B. dalzielli* (West Africa), and *B. papyrifera* (Northeast Africa) grouped in the cluster A are clearly different with Bos_i_ between 7000 and 310,000 (Table 2). The species *B. dalzielli* (cluster A) exhibit rather high Bos_i_ values, between 130,000 and 305,000. Frankincense with low and very low PTA concentrations like *B. frereana*, *B. rivae*, and *B. neglecta* (from Kenya) exhibit Bos_i_ between 0 and 5. Samples of the species *B. carterii* and *B. neglecta* (from Somalia) assembled in cluster D show Bos_i_ between 75 and 900. Hence, Bos_i_ might be a rather convenient tool to classify *Boswellia* sample based on just three components. Yet for more accurate classification, the entire PTA composition of the oleogum resin needs to be investigated.

### 2.5. PTA Composition of Boswellia occulta Oleogum Resin

The method developed here and their statistical evaluation was also used to analyze a recently discovered *Boswellia* species, *B. occulta*. This species is distributed in a small area in Somaliland in northwestern Somalia and was first described by Thulin et al. in 2019 [16]. The extract obtained from the oleogum resin of *B. occulta* had a white, crystalline form with an extraction yield of 66.3% (*w*/*w*). PTA analysis by HPLC-MS/MS exhibited concentrations of 2.667 µg KBA, 4.386 µg LA, 6.991 µg α-BA, 23.854 µg β-BA, 33.644 µg AKBA, 15.389 µg ALA, 16.466 µg α-ABA, and 30.198 µg β-ABA, each per milligram oleogum resin yielding a total PTA content of 13.4% (*w*/*w*) in the *B. occulta* oleogum resin. Calculation exhibited a Bos_i_ of 23,059 for *B. occulta*. Based on these results, the sample showed similarity to oleogum resins of the species *B. sacra*, especially to samples of the type Black Hojari (sample # 2: Bos_i_ = 35,612 and sample # 6: Bos_i_ = 13,137). Moreover, the PTA composition and Bos_i_ of the sample resemble oleogum resins of the species *B. papyrifera*, especially those from Ethiopia and Sudan (sample # 15: Bos_i_ = 34,172 and sample # 17: Bos_i_ = 32,469). Furthermore, by using the component coefficients calculated from PCA, the score for *B. occulta* was determined and added subsequently to the biplot (Figure 4B). This illustrated additionally the similarity of *B. occulta* to *B. sacra* and *B. papyrifera* regarding their PTA composition. For a more specific differentiation between these three species, determination of their essential oil compositions should be considered [14,29,30].

### 2.6. Cytotoxicity of Frankincense Extracts towards Triple Negative Human Breast Cancer Cells

**Figure 5:**
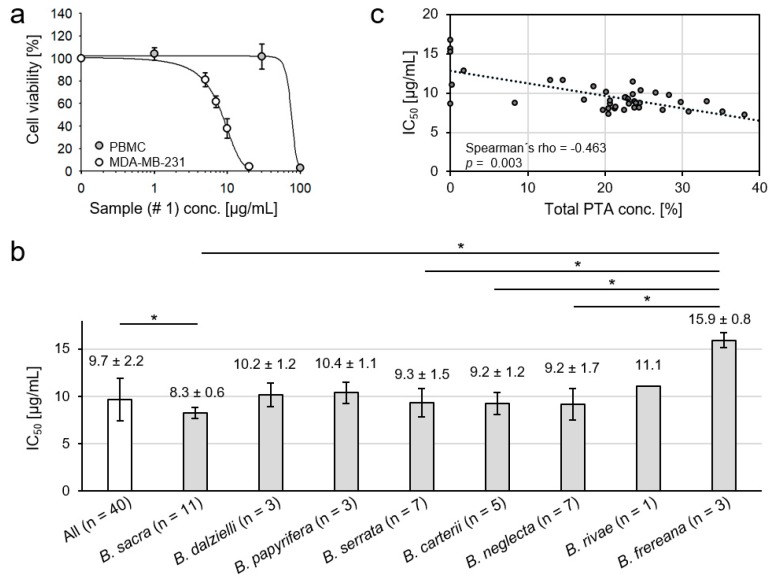
Cytotoxicity of frankincense extracts against the triple negative metastatic human breast cancer cell line MDA-MB-231. (**a**) Cancer cells and peripheral blood mononuclear cells (PBMC) were treated for 72 h with *B. sacra* extract (sample # 1) and cell viability was analyzed by XTT assay (mean ± SEM, *n* = 3). The extract inhibits selectively the viability of MDA-MB-231 cells, PBMC are relatively resistant to the extract. (**b**) Significant correlation between total concentration of PTA in the frankincense extracts (*w*/*w*) and the cytotoxicity towards breast cancer cells (Spearman’s rank test, *p* = 0.003). (**c**) Comparison of cancer cell toxicity of various *Boswellia* extracts. Groups were compared by Kruskal-Wallis one-way analysis of variances and Mann-Whitney U test, data are mean ± SD, * *p* < 0.05.

An extract from *B. sacra* oleogum resin inhibited concentration-dependently the viability of treatment-resistant MDA-MB-231 human breast cancer cells, whereas normal peripheral blood mononuclear cells (PBMC) from healthy volunteers consisting primarily of lymphocytes and monocytes were much less sensitive to the extract providing evidence for selectivity against cancer cells (Figure 5a). Hence, all forty *Boswellia* oleogum resin extracts have been investigated for their toxicity against MDA-MB-231 breast cancer cells and the half maximal inhibitory concentrations (IC_50_) were determined. The extracts exhibited considerable cytotoxicity towards MDA-MB-231 cells with an average IC_50_ of 9.7 µg/mL (Figure 5b). Moreover, extracts obtained from oleogum resins of *B. sacra* exhibited the highest toxicity with an IC_50_ of 8.2 µg/mL (Mann-Whitney U test: *p* = 0.015). Although extracts obtained from resins of *B. frereana* showed significantly lower cytotoxicity compared to *B. sacra* (Mann-Whitney U test: *p* = 0.013), *B. serrata* (Mann-Whitney U test: *p* = 0.023), *B. carterii* (Mann-Whitney U test: *p* = 0.037), and *B. neglecta* (Mann-Whitney U test: *p* = 0.023), their toxicity against cancer cells is considerable and should not to be neglected (Figure 5b). This indicates that, besides the PTA that were investigated by us, *Boswellia* oleogum resins must contain additional substances toxic for cancer cells, which merit further investigation.

Still, total amounts of PTA correlate significantly to extract toxicity to cancer cells (Figure 5c). Analysis of the correlation between the IC_50_ values and concentrations of the individual substances revealed the highest positive correlation between cytotoxicity and the content of acetylated PTA without keto groups. Thus, the β-ABA exhibited the highest Spearman’s correlation between cytotoxicity and its content in oleogum resin (*p* = 0.0001), followed by ALA (*p* = 0.0004), α-ABA (*p* = 0.002), LA (*p* = 0.008), and β-BA (*p* = 0.022). Likewise, the extract obtained from sample # 26 (*B. carterii*) exhibited the highest content of β-ABA und ALA and high levels of α-ABA and showed the highest toxicity against the tested cancer cells with an IC_50_ = 7.28 µg/mL.

Doxorubicin, a chemotherapeutic agent for treatment of patient with breast cancer, was used as a positive control and exhibited an approximately twentyfold higher toxicity with an IC_50_ = 0.41 µg/mL ± 0.03 µg/mL. However, severe adverse effects of doxorubicin have been reported, such as cardio- and nephrotoxicities [31,32]. In contrast, for preparations from frankincense only minimal adverse events like heartburn or nausea have been reported [33].

Polyphenols are another group of natural compounds exhibiting cytotoxic efficacy against breast cancer cells [34,35]. In a previous study, we have demonstrated that an Artemisia annua extract containing polyphenols, such as casticin or chrysosplenol D, likewise exhibits cytotoxicity against MDA-MB-231 cells with an IC_50_ of 18.2 µg/mL [36]. Hence, the PTA-containing *Boswellia* extracts are more effective against breast cancer cells compared to a polyphenol-containing Artemisia annua extract.

### 2.7. Cytotoxicity of Different Extraction Fractions Obtained from Oleogum Resins of the Species Boswellia sacra

**Figure 6:**
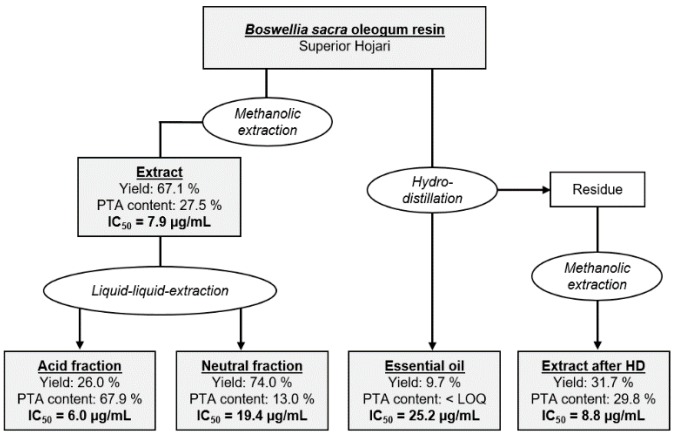
Fractionated extraction of *Boswellia sacra* oleogum resin (sample # 1). Yields refer to the previous extraction stage, respectively. Concentrations are total amounts of PTA in percent (*w*/*w*). Cytotoxicity was analyzed by the XTT cell viability and proliferation assay (MDA-MB-231, 72 h, *n* = 3).

In addition to PTA, *Boswellia* oleogum resins contain further ingredients toxic to breast cancer cells. These might be neutral pentacyclic triterpenic substances like amyrin and lupeol, or mono- and bicyclic monoterpenes like limonene and α-pinene [14,18,37]. Particularly, *B. frereana* contains high amounts of 3-epi-lupeol [12], which could explain its toxicity against the MDA-MB-231 breast cancer cells in the absence of PTA. Also, other studies indicated that the activity of *Boswellia* oleogum resin extracts can be higher than that of purified individual boswellic acids [3]. Therefore, oleogum resin of the species *B. sacra* (sample # 1) was separated into different fractions (Figure 6 and Table S2). After extraction with methanol, the extract was separated by liquid-liquid-extraction yielding an acid fraction and a neutral fraction. In a different approach, the oleogum resin was hydrodistilled to obtain the essential oil. Afterwards, the residue was extracted with methanol yielding an extract after hydrodistillation. By enrichment of acidic components, the total PTA concentration increased from 27.5% to 67.9%. The enriched acid fraction exhibited a higher cytotoxicity with an IC_50_ of 6.0 µg/mL compared to the non-fractionated extract with an IC_50_ of 7.9 µg/mL (Student´s t-test: *p* = 0.012). The neutral fraction with decreased PTA concentration and the essential oil that was void of detectable PTA, both exhibited considerably lower cytotoxicity with an IC_50_ = 19.4 µg/mL and an IC_50_ = 25.2 µg/mL for the neutral fraction and the essential oil, respectively. Although the PTA contents of the extract after hydrodistillation could be increased by removal of the essential oil, it showed lower cytotoxicity with an IC_50_ of 8.8 µg/mL compared to the non-fractionated extract. It is possible that high temperatures during hydrodistillation degrade natural emulsifiers. As a result, solubility of the nonpolar PTA and thereby the availability for the cells could be decreased. In line with that, the bioavailability of boswellic acids in vivo depended critically on the presence of bile acids and was therefore significantly increased by a concomitant high-fat meal [38]. Likewise, bioavailability of boswellic acids in a formulation with lecithin, a natural emulsifier, was greatly enhanced [39]. The possible synergetic effects of acidic components and components of the essential oil could not be disregarded either.

Hence, acidic components from the methanolic extract exhibit the highest cytotoxicity toward breast cancer cells. Only eight acidic PTA components were investigated in this study, while other similar compounds such as tirucallic acids [9,10,40,41] deserve further investigation. *Boswellia* oleogum resin also contains dehydro boswellic acids. However, compared to PTA investigated here, only small quantities of dehydro boswellic acids were detected in *Boswellia* oleogum resins [9,10]. Hence, according to current knowledge, primarily acetylated PTA represent a cytotoxic principle of the *Boswellia* oleogum resins. In the future, the group of acetylated acidic components in frankincense deserves more precise examination.

## 3. Materials and Methods

### 3.1. Plant Material

Frankincense oleogum resins were purchased from competent trustworthy traders or were obtained from cooperation partners. Samples # 1–8, 12–15, 17–21, and 25–40 were purchased from Georg Huber (Jeomra, Seeheim, Germany). Samples # 22–24 were from Alfred Galke (Alfred Galke GmbH, Bad Grund, Germany). Samples # 9–11 were provided by Luay J. Rashan (Dhofar University, Oman) and sample # 16 was from Stephan Pohl (Staufen, Germany). A sample of *B. occulta* was provided by Mats Thulin (Evolutionary Biology Centre, Department of Organismal Biology, Uppsala University, Sweden) [16]. Voucher specimens of all *Boswellia* oleogum resins are deposited at the Herbarium of the Botanical Garden of Ulm University, Institute of Systemic Botany and Ecology, Germany (voucher: ULM-24224, see Table S1).

### 3.2. Materials

All solvents and chemicals were of analytical reagent grade. The solvents used for the extraction and for HPLC-MS/MS analysis were methanol, acetic acid (both HiPerSolv Chromanorm, VWR chemicals, Fontenay-sous-Bois, France), and ultrapure water (reverse-osmosis type water (pureAqua, Schnaitsee, Germany) coupled to a Milli-Q station (Millipore, Eschborn, Germany)). The reference substances, acetyl-α-boswellic acid (α-ABA), acetyl-β-boswellic acid (β-ABA), α-boswellic acid (α-BA), β-boswellic acid (β-BA), acetyl-11-keto-β-boswellic acid (AKBA), 11-keto-β-boswellic acid (KBA), and maslinic acid (MA) were purchased from Extrasynthese (Genay Cedex, France). Acetyl-lupeolic acid (ALA) and lupeolic acid (LA) were isolated and characterized as previously published [7,8,10].

### 3.3. Extraction Procedure

For extraction, the frankincense oleogum resins were cooled down to −20 °C and ground. 10 g of freshly ground resins were extracted with 40 mL methanol at room temperature for 45 min with continuous stirring. After centrifugation (5 min at 5000× *g*), the supernatant was collected and the extraction was repeated twice. The combined supernatants were filtered through regenerated cellulose and evaporated to dryness by using a rotary evaporator yielding frankincense extract.

To obtain the acid and the neutral fractions, 5 g of *B. sacra* extract (sample # 1) were dissolved in 50 mL aqueous 2% KOH followed by extraction with ethyl acetate (5 × 25 mL). The combined ethyl acetate phases were dried with anhydrous Na_2_SO_4_ overnight and then evaporated to dryness yielding the neutral fraction (3.7 g). The aqueous phase was acidified with 1 M HCl to pH = 5 and extracted with ethyl acetate (5 × 25 mL). Likewise, the combined ethyl acetate phases were dried with anhydrous Na_2_SO_4_ overnight and then evaporated to dryness yielding the acid fraction (1.3 g).

To obtain essential oil, 100 g *B. sacra* oleogum resin (sample # 1) was freshly ground, added to 250 mL water, and mixed for several min until a thick homogenous dispersion was formed. To perform hydrodistillation, the mixture was heated for 7 h in an oil bath at 120 °C with continuous stirring. Afterwards, the obtained essential oil (9.7 g) was carefully separated from the aqueous phase in a separating funnel. After hydrodistillation, the residue was extracted with methanol as described earlier.

### 3.4. HPLC-MS/MS Analysis

The HPLC-MS/MS analysis was performed on an Agilent 1260 Infinity system (Agilent, Santa Clara, CA, USA) coupled with an AB API 2000 triple quadrupole mass spectrometer (Applied Biosystems, Foster City, CA, USA) using an electrospray ionization source (ESI). The data were obtained and processed by means of Analyst 1.6.1 software (AB Sciex, Framingham, MA, USA).

The chromatographic separation was performed using an analytical reversed-phase HPLC column (Dr. Maisch ReproSil-Pur Basic-C18 HD, 3 μm, 125 × 3 mm; Dr. Maisch GmbH, Ammerbruch, Germany) with a precolumn (Dr. Maisch ReproSil Universal RP, 5 μm, 10 × 4 mm). The flow rate was set to 600 μL/min and the injection volume was 20 μL. The mobile phase consisted of eluent A, methanol/water (80/20, *v*/*v*) acidified with 0.2% acetic acid, and eluent B, methanol acidified with 0.2% acetic acid. Initial conditions were 60% eluent A and 40% eluent B followed by a linear gradient to 90% eluent B over 15 min, then 90% eluent B until 25 min. Thereafter, followed a linear gradient to initial conditions until 25.5 min and reequilibration continued until 30 min. In order to stabilize the chromatographic system, the column was kept at a temperature of 28 °C.

MS/MS analysis was performed in the negative atmospheric pressure electrospray ionization mode and the multiple-reaction monitoring (MRM) detection mode. The ion source was heated to 300 °C and the ion spray voltage was set to −4250 V. Collision energy was optimized to −40 V. The dwell time for the MRM was 500 ms. The precursor ion at *m*/*z* 469.4 and the product ion of the highest intensity at *m*/*z* 391.4 were selected for KBA. Similarly, the ions at *m*/*z* 511.4 and 59.0 were used for AKBA, the ions *m*/*z* 455.4 and 437.2 for α-BA, β-BA and LA, the ions *m*/*z* 497.2 and 59.0 for α-ABA, β-ABA and ALA and the ions *m*/*z* 471.4 and 423.0 for MA. Quantifications of the analyte concentrations of α-ABA, β-ABA, α-BA, β-BA, AKBA, KBA, ALA, and LA, were done using the external calibration method and maslinic acid (MA) as internal standard. Extract samples were dissolved in methanol and spiked with internal standard for sample preparation. To determine the analyte concentrations in oleogum resins, the results of the extract analysis were corrected by extraction yields. The internal standard was used to compensate possible equipment conditioned variabilities. Furthermore, the internal standard enables further applications, such as analysis of clinical matrices (e.g., human plasma). Absence of maslinic acid in frankincense was evaluated by analysis of samples of all investigated *Boswellia* species (LOD = 3 ng MA/mg extract).

### 3.5. Validation of the HPLC-MS/MS Method

The HPLC-MS/MS method was validated in terms of linearity, precision, accuracy, limit of detection (LOD) and limit of quantification (LOQ). To obtain the linearity and to determine LOD and LOQ, standard solutions in the range from 10 ng/mL to 1000 ng/mL (8 levels) were analyzed, each in triplicates. The regression, LOD and LOQ were calculated with Valoo software (Applica, Bremen, Germany) based on the standardization criteria of DIN 32645 as defined by the German standardization committee [42]. For evaluation of accuracy, the recovery was determined by using the standard addition method. Hence, three samples were spiked at three levels and analyzed, each in triplicates. Precision was determined by analysis of reference standards at three levels with six replicates on four different days yielding the intraday variations and the interday variations. The results of the validation are shown in Table 1.

### 3.6. Analysis of Cancer Cell Cytotoxicity

Stock solutions were prepared in dimethyl sulfoxide (DMSO) and were further diluted with medium, supplemented with 1% fetal calf serum (FCS). The final DMSO concentration in the medium was 0.5% for all experiments. Treatment-resistant MDA-MB-231 breast cancer cells from Cell Biolabs (San Diego, CA, USA) were cultured in Dulbecco´s Modified Eagle Medium (DMEM, 4.5 g/L glucose, GlutaMax; Life Technologies, Carlsbad, CA, USA) supplemented with 10% FCS, 0.1 mM MEM nonessential amino acids, 100 U/mL penicillin, and 100 mg/mL streptomycin and passaged twice a week at a density of 5000 cells/cm^2^. Peripheral blood mononuclear cells (PBMC) isolated from whole venous blood of healthy volunteers by density gradient centrifugation by using Biocoll (Biochrom GmbH, Berlin, Germany) were cultured in RPMI 1640 medium, 2 mM L-glutamine (Life Technologies) supplemented with 10% FCS and penicillin/streptomycin. The study using human blood was approved by the institutional Ethic Committee and the volunteers gave their formal consent. Cells were cultured in a humidified incubator at 37 °C and 5% CO_2_ atmosphere. For the experiments, 3000 MDA-MB-231 cells or 300,000 PBMC were seeded per well into 96-well plates in medium supplemented with 10% FCS overnight. Next day, cells were treated with different concentrations of the extracts, pure compounds, or 0.5% DMSO vehicle for 72 h by using a Tecan D300e Digital Dispenser (Tecan, Männedorf, Switzerland). Cell viability was analyzed after addition of 2,3-Bis-(2-methoxy-4-nitro-5-sulfophenyl)-2H-tetrazolium-5-carboxanilide salt (XTT). Absorbance of the orange formazan dye formed by reduction of the tetrazolium salt by mitochondrial dehydrogenases of viable cells was measured using an Infinite M1000 PRO Tecan plate reader at 450 nm with a 630 nm reference filter. Quantification of the viability was accomplished by subtracting a blank value containing the respective extract and by normalization to the vehicle control.

### 3.7. Statistical Analysis

Each experiment was repeated three times and the data are expressed as the mean ± standard deviation (SD) or standard error of the mean (SEM) as indicated. Statistical analysis was performed using Minitab 18 software (Minitab, Munich, Germany). All data were tested for normal distribution by the Anderson-Darling test and equality of variances by Levene’s test. Sample groups were compared by one-way ANOVA and the Student´s t-test for parametric data and the Kruskal-Wallis one-way analysis of variance and the Mann-Whitney U test for non-parametric data. Due to the small sample size, the sample of the species *B. rivae* was excluded from comparison of groups (*n* = 1). Correlations for non-parametric data were investigated by Spearman´s rank correlation. Cluster analysis was performed with hierarchical agglomerative clustering, Ward´s method, and squared Euclidean distances. Principal component analysis (PCA) was derived from a covariance matrix of the data.

## 4. Conclusions

A highly sensitive, selective, and accurate method for the determination of eight pentacyclic triterpenic acids in oleogum resins of the genus *Boswellia* by HPLC-MS/MS has been developed and validated. Forty-one different frankincense samples of nine different *Boswellia* species were extracted and analyzed. Hence, patterns in the composition of the pentacyclic triterpenic acids were discovered assisting discrimination among different *Boswellia* species and geographic growth locality. Moreover, this study provides evidence for the cytotoxic efficacy of frankincense extracts towards treatment-resistant human breast cancer cells in vitro. The cytotoxic activity correlates significantly with the pentacyclic triterpenic acid contents in the extracts. Enrichment of the acidic component fraction containing pentacyclic triterpenic acids increased the extract’s cytotoxicity. Thus, acid fractions of the *Boswellia* oleogum resin extracts deserve further studies aiming at the development of new anticancer drugs.

## Figures and Tables

**Table 1:** HPLC-MS/MS validation data: calibration curves (with internal standard), limit of detection and limit of quantification, evaluation of precision, and recovery test for evaluation of accuracy.

Compound	Regression Equation ^1^			LOD ^2^ [ng/mg]	LOQ ^2^ [ng/mg]	Intraday and Interday Precision ^3^ (RSD [%])						Recovery ^4^ [%]	
	yayi=a×(caci)+b					Low Level		Mid Level		High Level			
	Slope (a)	Offset (b)	R^2^			Intraday	Interday	Intraday	Interday	Intraday	Interday	Mean	SD
KBA	8.5009	0.0207	0.9992	0.7	2.6	7.6	6.8	6.4	2.0	7.0	4.5	97.7	4.9
LA	2.0181	0.0437	0.9984	1.9	7.2	9.3	6.8	5.8	5.1	9.8	4.8	99.8	3.6
α-BA	2.9393	0.0344	0.9997	1.0	6.0	9.9	7.4	7.2	7.4	7.7	3.8	91.2	4.6
β-BA	2.5022	0.0246	0.9997	1.6	5.9	6.4	6.3	5.9	4.7	7.1	3.9	95.1	1.9
AKBA	34.9050	0.1357	0.9998	0.4	1.5	9.4	7.9	5.8	6.4	7.8	2.3	94.7	5.0
ALA	20.5466	0.3225	0.9989	1.6	6.0	7.6	6.4	5.0	7.4	6.3	2.3	97.7	2.5
α-ABA	36.4455	0.4111	0.9998	0.9	3.5	6.9	6.0	5.4	6.4	7.5	2.1	98.7	1.5
β-ABA	37.0869	0.4314	0.9999	0.9	3.4	5.7	6.0	5.4	5.7	6.1	2.1	97.7	3.2

^1^*y_a_* refers to the peak area [cps] of the corresponding compound; *y_i_* refers to the peak area [cps] of the internal standard; *c_a_* refers to the concentration [ng/mL] of the corresponding compound; *c_i_* refers to the concentration [ng/mL] of the internal standard. ^2^ LOD and LOQ according to DIN 32645 and a corresponding sample concentration of 10 mg/mL yielding concentrations of PTA in ng/mg of methanolic dry extract (*w*/*w*). ^3^ Low level = 75 ng/mL; mid level = 200 ng/mL; high level = 500 ng/mL; intraday *n* = 6; interday *n* = 4. ^4^ Method of standard addition; 3 samples (# 2, 12, and 22); spiked on three levels each (100 ng/mL, 200 ng/mL, and 300 ng/mL).

**Table 2:** Concentrations of pentacyclic triterpenic acids (PTA) in *Boswellia* oleogum resins and extract cytotoxicity in terms of IC_50_ values to MDA-MB-231 breast cancer cells (72 h, *n* = 3). *Boswellia* index (Bos_i_) is a parameter based on the PTA composition for identification of *Boswellia* species.

Samples				Yield of Extraction (*w*/*w*) [%]	Concentrations of PTA in *Boswellia* oleogum Resins									IC_50_ [µg/mL]		Bos_i_
					Deacetylated PTA [µg/mg]				Acetylated PTA [µg/mg]				Σ PTA [%]	MDA-MB-231		
#	Species	Specification	Origin		KBA	LA	α-BA	β-BA	AKBA	ALA	α	β-ABA		Mean	SEM	
1	*B. sacra*	Superior Hojari	Oman	67.1	2.716	4.779	10.333	27.242	41.819	19.794	29.121	48.596	18.4	7.90	0.31	181,631
2	*B. sacra*	Black Hojari	Oman	64.2	2.834	4.683	7.304	20.174	29.178	17.971	17.292	30.624	13.0	8.70	1.20	35,612
3	*B. sacra*	Royal Hojari	Oman	63.1	1.221	2.566	4.142	13.110	34.373	16.908	20.736	35.942	12.9	8.08	0.22	168,427
4	*B. sacra*	Royal Hojari	Oman	57.7	1.101	5.810	6.437	21.131	32.909	29.762	25.259	49.536	17.2	8.84	0.36	216,446
5	*B. sacra*	Red Hojari	Oman	62.8	2.162	7.787	13.283	29.391	35.891	31.809	38.498	62.284	22.1	7.67	0.25	307,249
6	*B. sacra*	Black Hojari	Oman	49.7	2.294	3.256	7.668	24.805	21.773	10.203	16.939	31.230	11.8	8.99	0.07	13,137
7	*B. sacra*	Royal Hojari	Oman	53.6	1.616	4.067	7.635	22.360	37.469	26.093	27.182	38.505	16.5	7.62	0.69	98,912
8	*B. sacra*	Superior Hojari	Oman	61.1	2.100	4.196	6.705	18.180	33.344	25.456	18.955	32.406	14.1	8.63	1.16	68,063
9	*B. sacra*	Najdi	Oman	60.6	0.996	3.954	5.843	19.123	26.083	24.321	23.123	44.474	14.8	8.14	0.37	130,306
10	*B. sacra*	Hojari	Oman	59.3	1.087	2.950	4.651	18.614	21.776	13.042	18.792	39.895	12.1	7.33	0.26	74,933
11	*B. sacra*	Sahli	Oman	56.0	1.153	3.025	4.626	13.801	30.058	24.080	23.679	36.737	13.7	8.81	0.92	138,655
**Mean**				**59.6**	**1.753**	**4.279**	**7.148**	**20.721**	**31.334**	**21.767**	**23.598**	**40.930**	**15.2**	**8.25**		
*SD*				*5.0*	*0.691*	*1.501*	*2.690*	*5.068*	*6.330*	*6.788*	*6.366*	*9.611*	*3.1*	*0.57*		
12	*B. dalzielli*	Janawhi	Burkina Faso	73.8	11.663	5.152	13.250	17.384	53.425	14.117	27.349	31.446	17.4	11.44	0.82	134,345
13	*B. dalzielli*	-	Nigeria	68.5	9.859	9.334	16.226	20.266	72.197	21.618	39.248	37.964	22.7	8.99	0.58	303,439
14	*B. dalzielli*	-	Senegal	72.2	13.859	5.276	16.121	16.794	68.332	13.450	29.715	28.115	19.2	10.10	1.21	142,477
**Mean**				**71.5**	**11.794**	**6.588**	**15.199**	**18.148**	**64.652**	**16.395**	**32.104**	**32.508**	**19.7**	**10.18**		
*SD*				*2.7*	*2.003*	*2.379*	*1.689*	*1.858*	*9.913*	*4.536*	*6.299*	*5.010*	*2.7*	*1.22*		
15	*B. papyrifera*	1st Quality	Ethiopia	65.7	4.384	3.689	10.920	23.645	43.941	10.575	21.379	30.049	14.9	9.47	0.09	34,172
16	*B. papyrifera*	-	Eritrea	66.4	3.406	2.375	5.549	16.434	27.844	6.591	14.259	19.871	9.6	11.65	1.03	7196
17	*B. papyrifera*	-	Sudan	70.0	3.187	3.033	10.331	31.762	16.809	9.175	20.171	46.505	14.1	10.12	0.80	32,469
**Mean**				**67.4**	**3.659**	**3.032**	**8.933**	**23.947**	**29.531**	**8.780**	**18.603**	**32.142**	**12.9**	**10.41**		
*SD*				*2.3*	*0.638*	*0.657*	*2.946*	*7.668*	*13.645*	*2.021*	*3.810*	*13.439*	*2.8*	*1.12*		
18	*B. serrata*	1st Quality	India	59.1	5.330	4.949	16.373	49.706	11.324	6.055	11.693	36.453	14.2	8.81	0.85	−9953
19	*B. serrata*	1st Quality	India	62.0	22.290	10.157	25.308	49.748	18.692	6.581	11.703	30.560	17.5	9.81	1.39	−21,112
20	*B. serrata*	1st Quality	India	63.2	3.091	4.299	15.560	47.921	13.644	7.748	16.170	41.879	15.0	8.14	0.72	−6994
21	*B. serrata*	-	India	57.1	5.793	5.548	13.212	54.020	8.561	3.867	7.420	23.143	12.2	8.29	0.87	−8884
22	*B. serrata*	cut	India	58.4	9.797	5.589	15.345	42.671	13.257	5.069	10.118	29.361	13.1	7.82	0.15	−9787
23	*B. serrata*	ground	India	65.8	6.256	2.493	8.924	28.286	9.253	3.440	6.181	19.756	8.5	11.62	0.53	−2909
24	*B. serrata*	ground	India	55.6	4.047	3.567	12.167	38.301	8.068	4.635	8.421	23.630	10.3	10.89	0.96	−4614
**Mean**				**60.2**	**8.086**	**5.229**	**15.270**	**44.379**	**11.828**	**5.342**	**10.244**	**29.255**	**13.0**	**9.34**		
*SD*				*3.6*	*6.610*	*2.440*	*5.102*	*8.771*	*3.745*	*1.539*	*3.346*	*7.875*	*3.0*	*1.47*		
25	*B. carterii*	1st Quality	Somalia	61.1	0.080	10.850	11.829	38.053	0.103	23.494	19.450	46.412	15.0	10.38	0.65	−779
26	*B. carterii*	1st Quality	Somalia	66.7	0.016	15.168	15.501	57.312	0.060	47.071	33.760	84.567	25.3	7.28	0.18	−5376
27	*B. carterii*	green frankincense	Somalia	49.4	0.143	15.406	28.861	66.660	0.001	0.073	0.014	0.130	11.1	9.42	0.25	75
28	*B. carterii*	1st Quality	Somalia (Puntland)	59.8	0.865	19.782	36.476	83.868	0.006	0.042	0.033	0.134	14.1	9.83	0.94	95
29	*B. carterii*	2nd Quality	Somalia (Puntland)	68.1	6.454	3.836	20.667	18.149	49.343	15.155	17.446	25.477	15.7	9.26	1.13	46,026
**Mean**				**61.0**	**1.512**	**13.008**	**22.667**	**52.809**	**9.903**	**17.167**	**14.140**	**31.344**	**16.3**	**9.23**		
*SD*				*7.4*	*2.784*	*6.022*	*10.023*	*25.475*	*22.048*	*19.518*	*14.341*	*35.506*	*5.4*	*1.17*		
30	*B. neglecta*	Muqlo (black)	Somalia	76.4	1.444	41.127	26.801	80.994	0.044	3.175	0.751	3.652	15.8	9.06	0.06	360
31	*B. neglecta*	Muqlo&Gunro	Somalia	84.4	1.565	35.831	26.739	75.827	0.143	10.829	2.618	13.088	16.7	7.89	0.86	888
32	*B. neglecta*	Muqlo (black)	Somalia	80.5	1.731	38.702	29.818	85.886	0.048	3.017	0.614	4.275	16.4	8.10	0.73	430
33	*B. neglecta*	Mix	Somalia (Puntland)	84.5	0.635	31.333	24.802	73.627	0.081	6.484	1.801	7.140	14.6	9.13	0.86	546
34	*B. neglecta*	Mirafur	Somalia (Bakool/Mudug)	49.4	0.240	7.711	7.217	20.613	0.025	1.997	0.490	2.613	4.1	8.73	1.53	71
35	*B. neglecta*	1st Quality (black)	Kenya	42.3	0.062	0.836	1.920	3.212	0.018	0.416	0.081	0.794	0.7	12.85	1.14	5
36	*B. neglecta*	1st Quality (black)	Kenya	68.3	<LOQ	<LOQ	<LOQ	<LOQ	0.002	<LOQ	<LOQ	0.004	0.0	8.63	0.60	0
**Mean**				**69.4**	**0.811**	**22.220**	**16.757**	**48.594**	**0.052**	**3.703**	**0.908**	**4.510**	**9.8**	**9.20**		
*SD*				*17.1*	*0.752*	*18.524*	*13.088*	*38.757*	*0.048*	*3.801*	*0.958*	*4.454*	*7.8*	*1.67*		
37	*B. rivae*	1st Quality	Ogaden	64.6	0.038	0.049	0.052	0.307	0.055	0.099	0.056	0.194	0.1	11.10	0.97	0
38	*B. frereana*	Maydi (Mujarwaal/Fas Kabir)	Somalia (Somaliland)	90.6	<LOQ	<LOQ	<LOQ	<LOQ	<LOQ	<LOQ	<LOQ	<LOQ	0.0	15.72	3.16	0
39	*B. frereana*	Maydi Mushaat	Somalia (Puntland)	87.0	<LOQ	<LOQ	<LOQ	<LOQ	<LOQ	<LOQ	<LOQ	<LOQ	0.0	16.81	3.54	0
40	*B. frereana*	Maydi Mujarwal	Somalia (Puntland)	88.8	<LOQ	<LOQ	<LOQ	<LOQ	<LOQ	<LOQ	<LOQ	<LOQ	0.0	15.28	0.93	0
**Mean**				**88.8**	**<LOQ**	**<LOQ**	**<LOQ**	**<LOQ**	**<LOQ**	**<LOQ**	**<LOQ**	**<LOQ**	**0.0**	**15.94**		
*SD*				*1.8*									*0.0*	*0.79*		

## Supplementary Materials

The following are available online, Table S1: Information on sources of *Boswellia* samples, concentrations of pentacyclic triterpenic acids (PTA) in *Boswellia* extracts, and vouchers specimens were deposited in the Herbarium of the Botanical Garden, Institute of Systemic Botany and Ecology, Ulm University; Table S2: Fractionated extraction of sample # 1 (*B. sacra*, Superior Hojari). Yields of extraction, concentrations of pentacyclic triterpenic acids (PTA), and toxicity to breast cancer cells MDA-MD-231 (72 h, *n* = 3).
