# Supplementary material for: Comparative Analysis of Pentacyclic Triterpenic Acid Compositions in Oleogum Resins of Different Boswellia Species and Their In Vitro Cytotoxicity against Treatment-Resistant Human Breast Cancer Cells
Source: Molecules. 2019 Jun 7;24(11):2153. doi: 10.3390/molecules24112153 (PMC6600171; doi:10.3390/molecules24112153)
Supplement: Supplementary file 1 [file molecules-24-02153-s001.pdf]

Supporting Information

Comparative Analysis of Pentacyclic Triterpenic Acid Compositions in Oleogum Resins of Different *Boswellia* Species and their In Vitro Cytotoxicity against Treatment-resistant Human Breast Cancer Cells

Michael Schmiech [1], Sophia J. Lang [1], Katharina Werner [1], Luay J. Rashan [2], Tatiana Syrovets [1] and Thomas Simmet [1,*]

[1] Institute of Pharmacology of Natural Products and Clinical Pharmacology, Ulm University, 89081 Ulm, Germany; michael.schmiech@uni-ulm.de (M.S.); sophia.lang@uni-ulm.de (S.J.L.); katharina.werner@uni-ulm.de (K.W.); tatiana.syrovets@uni-ulm.de (T.S.)

[2] Dhofar University, Research Center, Medicinal Plants Division, 211 Salalah, Sultanate of Oman; luayrashan@yahoo.com (L.J.R.)

* Correspondence: thomas.simmet@uni-ulm.de; Tel.: +49-731-500-65600 (Th.S.)

Table S1. Information on the sources of the *Boswellia* samples and the concentrations of pentacyclic triterpenic acids (PTA) in the *Boswellia* extracts; voucher specimens were deposited in the Herbarium of the Botanical Garden, Institute of Systematic Botany and Ecology, Ulm University.

Samples			Concentrations of PTA in *Boswellia* extracts									Herbarium specimens (ULM-24224)
			deacetylated PTA [µg/mg]				acetylated PTA [µg/mg]				Σ PTA [%]	
#	Supplier	Date of receipt	KBA	LA	α-BA	β-BA	AKBA	ALA	α-ABA	β-ABA		
1	Georg Huber	2017, 4th QTR	4.048	7.123	15.400	40.600	62.325	29.500	43.400	72.425	27.5	190425-1
2	Georg Huber	2017, 4th QTR	4.490	7.420	11.573	31.967	46.233	28.475	27.400	48.525	20.6	190425-2
3	Georg Huber	2017, 4th QTR	2.010	4.223	6.817	21.575	56.567	27.825	34.125	59.150	21.2	190425-3
4	Georg Huber	2018, 1st QTR	1.907	10.063	11.150	36.600	57.000	51.550	43,750	85.800	29.8	190425-4
5	Georg Huber	2018, 1st QTR	3.443	12.400	21.150	46.800	57.150	50.650	61.300	99.175	35.2	190425-5
6	Georg Huber	2018, 1st QTR	4.615	6.550	15.425	49.900	43.800	20.525	34.075	62.825	23.8	190425-6
7	Georg Huber	2018, 4th QTR	3.017	7.590	14.250	41.733	69.933	48.700	50.733	71.867	30.8	190425-7
8	Georg Huber	2018, 4th QTR	3.435	6.863	10.967	29.733	54.533	41.633	31.000	53.000	23.1	190425-8
9	Luay J. Rashan	2017	1.643	6.523	9.640	31.550	43.033	40.125	38.150	73.375	24.4	190425-9
10	Luay J. Rashan	2017	1.833	4.977	7.845	31.400	36.733	22.000	31.700	67.300	20.4	190425-10
11	Luay J. Rashan	2017	2.058	5.397	8.255	24.625	53.633	42.967	42.250	65.550	24.5	190425-11
**Mean**			**2.954**	**7,193**	**12.043**	**35.135**	**52.813**	**36.723**	**39.808**	**68.999**	**25.6**	
SD			*1.121*	*2.313*	*4.216*	*8.888*	*9.573*	*11.447*	*9.904*	*14.342*	*4,7*	
12	Georg Huber	2017, 4th QTR	15.800	6.980	17.950	23.550	72.375	19.125	37.050	42.600	23.5	190425-12
13	Georg Huber	2018, 2nd QTR	14.400	13.633	23.700	29.600	105.450	31.575	57.325	55.450	33.1	190425-13
14	Georg Huber	2018, 4th QTR	19.200	7.310	22.333	23.267	94.667	18.633	41.167	38.950	26.6	190425-14
**Mean**			**16.467**	**9.308**	**21.328**	**25.472**	**90.831**	**23.111**	**45.181**	**45.667**	**27.7**	
SD			*2.468*	*3.750*	*3.004*	*3.578*	*16.868*	*7.334*	*10.717*	*8.667*	*4.9*	
15	Georg Huber	2017, 4th QTR	6.675	5.617	16.625	36.000	66.900	16.100	32.550	45.750	22.6	190425-15
16	Stephan Pohl	2018	5.130	3.577	8.357	24.750	41.933	9.927	21.475	29.927	14.5	190425-16
17	Georg Huber	2018, 4th QTR	4.550	4.330	14.750	45.350	24.000	13.100	28.800	66.400	20.1	190425-17
**Mean**			**5.452**	**4.508**	**13.244**	**35.367**	**44.278**	**13.042**	**27.608**	**47.359**	**19.1**	
SD			*1.098*	*1.032*	*4.335*	*10.315*	*21.546*	*3.087*	*5.633*	*18.290*	*4.2*	
18	Georg Huber	2017, 4th QTR	9.025	8.380	27.725	84.167	19.175	10.253	19.800	61.725	24.0	190425-18
19	Georg Huber	2018, 2nd QTR	35.933	16.375	40.800	80.200	30.133	10.610	18.867	49.267	28.2	190425-19
20	Georg Huber	2017, 3rd QTR	4.893	6.807	24.633	75.867	21.600	12.267	25.600	66.300	23.8	190425-20
21	Georg Huber	2018, 4th QTR	10.150	9.720	23.150	94.650	15.000	6.775	13.000	40.550	21.3	190425-21
22	Alfred Galke	2017	16.775	9.570	26.275	73.067	22.700	8.680	17.325	50.275	22.5	190425-22
23	Alfred Galke	2015	9.510	3.790	13.567	43.000	14.067	5.230	9.397	30.033	12.9	190425-23
24	Alfred Galke	2018	7.273	6.410	21.867	68.833	14.500	8.330	15.133	42.467	18.5	190425-24
**Mean**			**13.366**	**8.722**	**25.431**	**74.255**	**19.596**	**8.878**	**17.017**	**48.660**	**21.6**	
SD			*10.598*	*3.953*	*8.180*	*16.124*	*5.810*	*2.396*	*5.207*	*12.489*	*4.9*	

Table S1. (continued)

25	Georg Huber	2017, 4th QTR	0.131	17.750	19.350	62.250	0.168	38.433	31.818	75.925	24.6	190425-25
26	Georg Huber	2018, 1st QTR	0.024	22.733	23.233	85.900	0.089	70.550	50.600	126.750	38.0	190425-26
27	Georg Huber	2018, 1st QTR	0.289	31.200	58.450	135.000	0.003	0.148	0.028	0.263	22.5	190425-27
28	Georg Huber	2018, 4th QTR	1.447	33.100	61.033	140.333	0.010	0.071	0.055	0.224	23.6	190425-28
29	Georg Huber	2018, 4th QTR	9.483	5.637	30.367	26.667	72.500	22.267	25.633	37.433	23.0	190425-29
**Mean**			**2.275**	**22.084**	**38.487**	**90.030**	**14.554**	**26.294**	**21.627**	**48.119**	**26.3**	
SD			*4.070*	*11.115*	*19.822*	*48.364*	*32.393*	*29.553*	*21.744*	*53.978*	*6.6*	
30	Georg Huber	2018, 2nd QTR	1.890	53.825	35.075	106.000	0.057	4.155	0.983	4.780	20.7	190425-30
31	Georg Huber	2018, 2nd QTR	1.853	42.433	31.667	89.800	0.170	12.825	3.100	15.500	19.7	190425-31
32	Georg Huber	2018, 4th QTR	2.150	48.067	37.033	106.667	0.060	3.747	0.763	5.310	20.4	190425-32
33	Georg Huber	2018, 4th QTR	0.751	37.067	29.340	87.100	0.096	7.670	2.130	8.447	17.3	190425-33
34	Georg Huber	2018, 4th QTR	0.486	15.600	14.600	41.700	0.050	4.040	0.991	5.287	8.3	190425-34
35	Georg Huber	2017, 4th QTR	0.147	1.975	4.535	7.585	0.042	0.983	0.192	1.875	1.7	190425-35
36	Georg Huber	2018, 4th QTR	< LOQ	< LOQ	< LOQ	< LOQ	0.003	< LOQ	< LOQ	0.006	0.0	190425-36
**Mean**			**1.040**	**28.424**	**21.750**	**62.693**	**0.068**	**4.774**	**1.166**	**5.886**	**12.6**	
SD			*0.902*	*22.263*	*15.208*	*45.731*	*0.052*	*4.327*	*1.095*	*5.026*	*9.1*	
37	Georg Huber	2017, 4th QTR	0.058	0.075	0.080	0.475	0.086	0.154	0.087	0.300	0.1	190425-37
38	Georg Huber	2017, 4th QTR	< LOQ	< LOQ	< LOQ	< LOQ	< LOQ	< LOQ	< LOQ	< LOQ	0.0	190425-38
39	Georg Huber	2018, 4th QTR	< LOQ	< LOQ	< LOQ	< LOQ	< LOQ	< LOQ	< LOQ	< LOQ	0.0	190425-39
40	Georg Huber	2018, 4th QTR	< LOQ	< LOQ	< LOQ	< LOQ	< LOQ	< LOQ	< LOQ	< LOQ	0.0	190425-40
**Mean**			**< LOQ**	**< LOQ**	**< LOQ**	**< LOQ**	**< LOQ**	**< LOQ**	**< LOQ**	**< LOQ**	**0.0**	
SD											*0.0*	
B.o.	Mats Thulin	2019, 2nd QTR	4.040	6.620	10.550	36.000	50.775	23.225	24.850	45.575	20.2	190425-41

Table S2. Fractionated extraction of sample # 1 (*B. sacra*, Superior Hojari). Yields of extraction, concentrations of pentacyclic triterpenic acids (PTA), and toxicity to breast cancer cells MDA-MD-231 (72 h, n = 3).

Fraction	Yield of extraction[1] (w/w) [%]	Concentrations of PTA									IC50 [µg/mL] MDA-MB-231	
		deacetylated PTA [µg/mg]				acetylated PTA [µg/mg]				Σ PTA [%]		
		KBA	LA	α-BA	β-BA	AKBA	ALA	α-ABA	β-ABA		mean	SEM
Acid fraction	26.0	10.100	12.850	34.400	89.700	155.000	74.350	120.000	183.000	67.9	6.03	0.29
Neutral fraction	74.0	0.967	3.190	5.140	20.600	28.100	16.700	17.600	37.400	13.0	19.36	2.33
Extract after hydrodistillation	31.7	4.470	7.400	16.200	37.000	69.050	36.450	48.200	79.400	29.8	8.76	1.10
Essential oil	9.7	< LOQ	< LOQ	< LOQ	< LOQ	< LOQ	< LOQ	< LOQ	< LOQ	0.0	25.15	3.49

[1] yields refer to respective extraction stage
